# Malignant Melanoma

**DOI:** 10.3390/healthcare2010001

**Published:** 2013-12-20

**Authors:** Eshini Perera, Neiraja Gnaneswaran, Ross Jennens, Rodney Sinclair

**Affiliations:** 1 Faculty of Medicine, Dentistry and Health Sciences, Melbourne University, Victoria 3010, Australia; E-Mails: eshinip@gmail.com (E.P.); neiraja.g@gmail.com (N.G.); 2 Epworth Dermatology, Suite 5.1, 32 Erin St, Richmond, Victoria 3121, Australia; 3 Epworth Healthcare, 32 Erin St, Richmond, Victoria 3121, Australia; E-Mail: jennens@bigpond.net.au

**Keywords:** melanoma, melanocyte, melanin, nevus, skin cancer, malignant melanoma, nodular melanoma, moles, cancer

## Abstract

Melanomas are a major cause of premature death from cancer. The gradual decrease in rates of morbidity and mortality has occurred as a result of public health campaigns and improved rates of early diagnosis. Survival of melanoma has increased to over 90%. Management of melanoma involves a number of components: excision, tumor staging, re-excision with negative margins, adjuvant therapies (chemo, radiation or surgery), treatment of stage IV disease, follow-up examination for metastasis, lifestyle modification and counseling. Sentinel lymph node status is an important prognostic factor for survival in patients with a melanoma >1 mm. However, sentinel lymph node biopsies have received partial support due to the limited data regarding the survival advantage of complete lymph node dissection when a micrometastasis is detected in the lymph nodes. Functional mutations in the mitogen-activated pathways are commonly detected in melanomas and these influence the growth control. Therapies that target these pathways are rapidly emerging, and are being shown to increase survival rates in patients. Access to these newer agents can be gained by participation in clinical trials after referral to a multidisciplinary team for staging and re-excision of the scar.

## 1. Introduction

Melanomas are malignant tumors derived from melanocytes. The most common site of involvement is the skin, although occasionally primary melanoma develops in other organs (eye, oral and nasal mucosa, vulval and anorectal mucosa, other gastrointestinal mucosa and the central nervous system (CNS)). 

Melanomas are a major cause of premature death from cancer. Recognized risk factors include personal or family history of melanoma, large numbers of naevi and/or dysplastic naevi, giant congenital melanocytic naevi, fair complexion, a tendency to sunburn, solar-damaged skin, a history of non-melanoma skin cancer, and immunodeficiency [1,2,3].

The most common sites for melanoma are the legs of women and the backs of men, despite these not being the sites of greatest sun exposure. Early detection is associated with improved survival [4].

Any malignancy will grow, grow irregularly, and function abnormally. Melanomas produce pigment in varying amounts and may elicit an immune response that will be reflected in the clinical appearance. Some melanomas may lack pigment. A small but significant number of melanomas are undiagnosable clinically. A history of change may be the only clue to the correct diagnosis (Table 1 and Table 2).

**Table 1:** Suggested patient history.

History	Significance
How long has the lesion been present?	Newly acquired lesions that persist for longer than one or two months may indicate neoplasm, particularly if the patient is in an older age group
Has a pigmented lesion changed in color or shape?	Alteration in shape or color may point towards malignancy
Has there been any bleeding?	Some benign lesions bleed, e.g., pyogenic granuloma or seborrhoeic keratosis. Basal cell carcinomas may also bleed. In general, melanomas bleed only when well advanced, and in such cases the diagnosis is usually obvious
Does the lesion itch?	Benign naevi or irritated seborrhoeic keratosis may itch when irritated by clothing, *etc*. While early melanomas are usually asymptomatic, some melanoma may develop an abnormal sensation that patients often find difficult to accurately describe
Is there a history of occupational sun exposure, or has the patient lived or worked in the tropics?	Skin cancers in general are related to life-time sun exposure. Malignant melanomas may be related to a single severe episode of sunburn, particularly in childhood
Is there a family history of skin cancer?	This may indicate a genetic susceptibility, inherited skin type or condition such as dysplastic naevus syndrome

**Table 2:** Characteristics of benign *vs*. malignant lesion.

Characteristic	Benign lesion	Potentially malignant lesion
Growth	Not growing	Growing
bleeding	absent	present
Number/location	Many other similar lesions	On sun exposed areas of the body
shape	Regular shape with smooth outline or line of symmetry	No symmetry
color	Uniform pigmentation	Variation in pigmentation within lesion
occurrence	Present for many years	New lesion

In 2009, the number of new cases of melanoma in Australia was 11,545 and the mortality figures for melanomas in 2010 were 1,452 [5]. Survival at five years following newly diagnosed invasive melanoma (Clark’s level 2–5) has increased from 87% in the 1980s to over 92% in the late 1990s [6] in Australia. The five-year survival rate continues to remain over 90% in 2010 [5]. In the absence of any new significant chemotherapy in that period, the improvement in survival has been attributed to public education and early diagnosis and excision [5]. Scar re-excision, sentinel lymph node biopsy, elective lymph node dissection, chemotherapy, radiotherapy and immunotherapy may improve survival at one year but have not been shown to improve five-year survival [7]. Adjuvant therapy with interferon results in a significantly greater disease-free survival rate although it is also associated with significant toxicity [8].

Macroscopic locoregional lymph node metastasis reduces five-year survival to 43% [9]. Patients who had locoregional metastasis had a better survival rate than those with metastasis to viscera [10]. The median survival of patients with metastasis to lung was 12 months while the medial survival of other visceral sites was seven months. Nonvisceral sites had a median survival of 18 months [11].

Functional mutations in genes in the mitogen-activated protein (MAP) kinase pathway are commonly detected in melanoma and these mutations influence growth control [12]. Several agents based on molecular understanding of this pathway have been approved for stage IV disease and additional agents are currently being evaluated in clinical trials. Various combinations of these agents are also being evaluated for stage IV disease and the BRAF inhibitor, dabrafenib is currently being evaluated in the U.S. in Phase III clinical trials in combination with trametinib in the adjuvant treatment of melanoma after surgical resection (COMBI-AD trial [NCT01682083]). The availability of adjuvant treatment, even in a trial setting, would necessitate a review of the current management of high-risk primary melanoma, and in particular the role of sentinel lymph node biopsy. 

Sentinel node biopsy is a technique performed immediately before surgical re-excision of the scar. It involves lymphatic mapping by lymphoscintigraphy and intraoperative injection of radioisotope and/or blue dye to identify the lymph node immediately downstream from the primary tumor. Sentinel lymph node status is the most important prognostic factor for disease-specific survival of patients with melanoma greater than 1 mm in thickness [13]. Histological examination of the first (“sentinel”) lymph node(s) identified with this technique has been demonstrated to identify the presence or absence of metastatic cells in the entire lymph node basin. This procedure is considered the most sensitive and specific staging test for the detection of micrometastatic melanoma in regional lymph nodes. 

Identification of micrometastatic disease in the sentinel lymph node is often followed by completion lymph node dissection (CLND). The Multicentre Selective Lymphadenectomy Trial (MLST-I, [NCT00297895]) demonstrated an overall survival advantage (78.3%) in patients who received a CLND after SLN was identified; compared to patients who were observed after a wide excision (73.1%) [14]. This has subsequently led to the MSLT II trial, to evaluate ultrasound observation of the SLND positive basin with delayed CLND if a recurrence is detected compared to immediate CLND. 

Data suggests that there are no additional benefits to CLND in patients with micrometastasis of less than 1 mm [15]. SLN is widely accepted in the U.S. based on the prognostic value and possibly contribution to the overall survival advantage. The procedure has received limited support in Australia, although results from the MSLT II trial may resolve much of this hesitancy.

Adjuvant chemotherapy is becoming an alternative to completion lymph node dissection for patients with a micrometastasis in the sentinel node. We suggest that patients with invasive melanoma on excision biopsy are referred to a rapid-access specialist multidisciplinary clinic for clinical staging (including sentinel node biopsy where indicated) and simultaneous re-excision of the scar.

## 2. Diagnosis of Melanoma and Benign Melanocytic Lesions

Melanoma is a histological diagnosis. Pigmented or non-pigmented skin lesions clinically suspicious of melanoma require biopsy and histological examination. Complex histology and in particular, spindle-cell morphology may require immunohistochemical stains in addition to routine H&E examination. The histological interpretation of pigmented skin lesions is not always straightforward and dermatopathology has emerged as a subspecialty discipline within pathology.

Benign melanocytic naevus is usually a clinical diagnosis. Good lighting is critical. If the physician is 100% confident of the diagnosis following visual inspection, then no further action is indicated. The one caveat to this is that a history of unstable morphology (change in size, shape or color) over several months would override the examination findings and is an indication for referral to a dermatologist or an excision biopsy. 

Where a diagnosis of benign naevus cannot be made with 100% certainty on visual inspection, and ipso facto cutaneous melanoma cannot be 100% excluded on clinical grounds, the patient should be referred to a dermatologist. Alternatively, the lesion could be excised and sent for a specialist pathologist opinion. Wherever possible, complete excision biopsy is preferred for diagnosis and formulation of a treatment plan. Complete excision of the suspicious lesion with a 2 mm lateral margin and down to fat is recommended. Only sample a lesion by punch or shave biopsy if complete excision is difficult (e.g., a large, facial pigmented lesion) because a biopsy may not be representative of the lesion as a whole, and it also alters the clinical appearance. Shallow shave biopsies should be avoided as prognosis hinges on full thickness tumor specimen.

The initial excision of a suspicious pigmented lesion is a diagnostic procedure. It is done to exclude or confirm melanoma. Thus, a benign histology does not mean that the procedure was unnecessary. If histology proves the lesion to be a melanoma, then definitive wider surgical excision and assessment for sentinel node biopsy is needed. This should be explained to the patient before the initial excision. 

Full-body photography on a single occasion can be useful in identifying new moles in patients. It is a helpful memory aid for the doctor and assists patients when performing self-mole checks. It is normal for occasional new moles to appear in adults until middle age. About one-third of melanomas appear in a pre-existing mole so it is more common for them to arise than from skin that appears normal. Thus, the appearance of a new mole in an adult flags the possibility of melanoma. In general, full-body photography is most useful in patients with a high mole count (>100) who find self-examination difficult. Serial photography with or without serial dermoscopy of individual moles will identify early changes suggestive of melanoma, but is generally reserved for patients with multiple atypical naevi.

A single photograph of a benign naevus is sometimes used to document and support the clinical decision not to biopsy that benign naevus. However, serial mole photography should not be used to diagnose benign melanocytic naevi. In other words, if the physician cannot 100% exclude a diagnosis of melanoma on visual inspection the appropriate diagnostic test is a biopsy and not serial photography.

## 3. Clinical Subtypes

There are several types of melanoma. Superficial spreading melanoma is the most common type of melanoma, usually presenting as an irregularly pigmented macule. About 50% have functional mutations in BRAF gene and 15%–25% mutations in NRAS gene [16]. Melanomas with NRAS mutations are more likely to be thicker tumors and to have a higher mitotic rate [17]. *In vitro*, melanoma cells with NRAS mutations are dependent on NRAS for survival and proliferation [17]. This would make NRAS an attractive therapeutic target in melanoma and once developed, pending an acceptable safety profile, chemotherapy agents that target NRAS could potentially make an important contribution to the future treatment of melanoma.

Nodular melanomas are aggressive tumors with an invasive growth pattern and can grow rapidly over weeks. They vary in color from black through to red and amelanotic, and frequently defy the ABCD rule. The mnemonic EFG standing for “elevated”, “firm” and “growing for more than one month” is more appropriate. They can be pedunculated. Often they are mistaken for a haemangioma or a pyogenic granuloma. About 50% have functional mutations in BRAF and 20% mutations in NRAS [18].

Acral lentiginous melanoma is the most common form of melanoma in the dark-skinned population. These are seen on the palms, soles or nail bed. Not all melanomas at these sites are of acral lentiginous type. Acral lentiginous melanomas commonly have functional mutation in the c-kit gene [18].

Lentigo maligna (Hutchinson’s melanotic freckle) is seen mostly on the face in sun-damaged elderly patients. They are a type of *in situ* melanoma with often a long delay before they become invasive. Patients will often be aware of these irregular, brown-to-black facial macules for many years. As such, they can be quite extensive at presentation even while still being restricted to the epidermis. Distinction from benign lentigo may be impossible without histology. Invasive melanoma or lentigo maligna melanoma (arising from a lentigo maligna) can sometimes complicate these lesions. Once this transition occurs it has the same behavior and prognosis as *de novo* invasive melanoma. Invasion can develop rapidly so excision is usually advised [19].

Desmoplastic melanoma is a rare and aggressive subtype of melanoma that usually comprises a superficial pigmented *in situ* melanoma overlying a poorly differentiated non-pigmented dermal spindle cell melanoma.

Amelanotic melanoma is the most difficult to diagnose clinically. These may present as a pink nodule or patch on the skin. On dermoscopy, a pigment network may be visible in some areas, but many are completely without pigment. 

Ocular melanoma is rare and often diagnosed late [20]*.* Uveal melanoma is associated with functional mutations in the *GNAQ*/*GNA11* genes [21,22]. These genes are not part of the ERK pathway. 

## 4. Treatment

There are 9 steps in the management of invasive melanoma of the superficial spreading and nodular sub-types. Subungual, mucosal, desmoplastic, ocular and acral lentiginous melanoma variants require management in a specialist center; further discussion of their management is beyond the scope of this article, as is the specific management of melanoma *in situ.*

### 4.1. Step 1: Excision of Primary Tumor

The majority of melanomas (70%) arise *de novo* in normal skin [23]. A smaller percentage (30%) arises within a pre-existing acquired naevus or a congenital naevus [23]. Prophylactic excision of acquired naevi is not recommended. Prophylactic excision of congenital naevi is considered for large naevi where a satisfactory cosmetic outcome is achievable. For large congenital naevi (>20 cm in diameter) including bathing-trunk naevi, an alternative to prophylactic excision is lifelong serial surveillance of the patient and serial excisional biopsy of suspicious nodules that may develop within the naevus. Excisional surgical biopsy with a lateral 2 mm margin of normal surrounding skin and a deep margin that includes the subcutis is recommended for all lesions suspected clinically of being melanoma. Where more than one lesion is excised, separate specimen bottles and accurate specimen labeling are essential. Shave biopsy is only acceptable when excisional biopsy is not feasible. Referral for specialist assessment is appropriate if the treating doctor is not comfortable performing the biopsy of a suspected melanoma. Punch biopsy risks sampling error when a melanoma arises within a benign naevus and is not generally recommended. Periodic clinical observation with or without photography is not recommended for suspected melanoma as it delays diagnosis. Baseline photography is useful in some patients with multiple naevi to identify new or changing moles that are suspicious of melanoma.

Clinical suspicion is based on the history of a new or changing pigmented lesion or a new, enlarging non-pigmented nodule. Visual inspection of the lesion is the most valuable tool to identify lesions that require excisional biopsy to exclude melanoma and distinguish from benign skin lesions. Visual inspection is enhanced by illumination, magnification and polarised light or oil immersion to reduce surface reflection. Epiluminescence microscopy can help distinguish melanoma from benign naevus, seborrhoeic keratosis, haemangioma and other benign tumors. Dermoscopy requires specific training [24]. It is important to recognise that not all melanomas will have distinctive signs on visual inspection or dermoscopy. In addition, dermoscopy is not required for the diagnosis of most melanomas.

Population screening accelerates melanoma diagnosis and improves patient survival. While feasible, the costs and potential savings of a universal screening program in Australia are not known. In the absence of this information, screening is currently limited to patients identified to be at higher risk of primary melanoma or to people who self-select for a skin check. 

High-risk patients that are usually advised to have regular screening include patients who have had one or more primary melanoma, patients with multiple non-melanoma skin cancer and some patients with multiple benign naevi. Many other at-risk patients, including those with a family history of melanoma, fair skin, red hair and freckles, also receive periodic screening. We developed informal guidelines on the appropriate frequency of skin checks for these people (Table 3).

**Table 3:** Assessment of skin cancer risk. At-risk patients are stratified by skin type, age and family history. A patient’s current risk level is determined by having at least one of the risk factors listed in the high, medium or low risk columns. For example, a patient may be considered high risk if they have a positive family history or if they have Type I skin and are over the age of 30. Risk levels can change with age and on detection of a melanoma or non-melanoma skin cancer and surveillance frequency should be adjusted accordingly.

	High risk	Medium risk	Low risk
Skin check frequency	Annual full-body skin checks recommended	One-off full-body skin check recommended with the frequency of re-examination required determined at initial skin check	Patient self-examination recommended
Type I skin with red hair	age over 30	age 20–29	below age 20
Type I skin without red hair	age over 40	age 30–39	below age 20
Type II skin	age over 60	age 40–59	below age 40
Type III skin	-	over 60	below age 60
Type IV and V skin	-	-	all ages
Family history	melanoma in first-degree relative	NMSC in first-degree relative	-
Past history	non-melanoma skin cancer (NMSC) or more than 20 solar keratosis	solar keratosis, multiple episodes of sunburn	-

Definitions: Type 1 skin: burns, never tans; Type II skin: burns, occasionally tans; Type III skin: tans, occasionally burns; Type IV skin: tans, rarely burns; Type V skin: never burns.

### 4.2. Step 2: Tumor Staging

Histological staging should be provided by the pathologist in the form of a synoptic report. Clinical staging includes palpation of the regional lymph node basins and examination for hepatomegaly. Sentinel lymph node biopsy should be discussed with all patients following diagnosis for invasive melanoma greater than 1mm thick, Clark level 4 melanomas, melanomas with more than two mitoses per high-power field, ulcerated melanoma or melanomas with significant regression, and melanomas of unknown malignant potential. Lymph node micrometastasis are genotyped, usually in the context of clinical studies, to determine whether it has acquired a BRAF (V600E) mutation. Mutation testing is usually confined to stage III clinical trials. As the results of clinical trials become available and the range of chemotherapeutic agents available to patients increase, these recommendations are likely to be modified further. In remote and regional centers, where patients may not have access to clinical trials or multidisciplinary care, the staging recommendations will vary. Additional baseline staging for node-positive patients may include an FBC, liver and renal function tests. A bone scan, CT scan, MRI or PET scan may also be required if the patient is to be enrolled in a clinical research trial. In the U.S. a full body CT and a MRI brain scan may be performed in the case of resected node positive prior to adjuvant treatment, however the advantage of using both imaging modalities concurrently is unknown. In Australia, there are no guidelines on the need for or frequency of repeat staging investigations in the absence of specific symptoms that suggest the presence of metastasis. In the United States (U.S.) the National Comprehensive Cancer Network (NCCN) guidelines delineates frequency of imaging that should be used [25]. There is limited evidence on whether the guidelines have resulted in early resection of stage IV cancer. The recommended staging nomenclature is the AJCC system (Table 4) [26]. 

Table 4Final version of 2009 AJCC melanoma staging and classification. TNM staging categories for cutaneous melanoma.

**Table:** 

Classification	Thickness (mm)	Ulceration status/mitoses
T		
Tis	NA	NA
T1	≤1.00	a: Without ulceration and mitosis <1/mm^2^
		b: With ulceration or mitoses ≥1/mm^2^
T2	1.01–2.00	a: Without ulceration
		b: With ulceration
T3	2.01–4.00	a: Without ulceration
		b: With ulceration
T4	>4.00	a: Without ulceration
		b: With ulceration

**Table:** 

	No. of metastatic nodes	Nodal metastatic burden
N		
N0	0	NA
N1	1	a: Micrometastasis ^*^
		b: Micrometastasis ^†^
N2	2–3	a: Micrometastasis ^*^
		b: Micrometastasis ^†^
		c: In transit metastases/satellites without metastatic nodes
N3	4+ metastatic nodes, or matted nodes, or in transit metastases/satellites with metastatic nodes

**Table:** 

	Site	Serum LDH
M		
M0	No distant metastases	NA
M1a	Distant skin, subcutaneous, or nodal metastases	Normal
M1b	Lung metastases	Normal
M1c	All other visceral metastases	Normal
	Any distant metastasis	Elevated

**Table:** 

	Clinical stage grouping			Pathological stage grouping		
	T	N	M	T	N	M
O	Tis	N0	M0	Tis	N0	M0
IA	T1a	N0	M0	T1a	N0	M0
IB	T1bT2a	N0N0	M0M0	T1bT2b	N0N0	M0M0
IIA	T2bT3a	N0N0	M0M0	T2bT3a	N0N0	M0M0
IIB	T3bT4a	N0N0	M0M0	T3bT4a	N0N0	M0M0
IIC	T4b	N0	M0	T4b	N0	M0
III	Any T Any T Any T	N1N2N3	M0M0M0			
IIIA				T1-4aT1-4a	N1aN2a	M0M0
IIIB				T1-4bT1-4bT1-4aT1-4aT1-4a/b	N1aN2aN1bN2bN2c	M0M0M0M0M0
IIIC				T1-4bT1-4bAny T	N1bN2bN3	M0M0M0
IV	Any T	Any N	M1	Any T	Any N	M1

NA = not applicable; LDH = lactate dehydrogenase; ^*^ Micrometastasis is diagnosed after sentinel lymph node biopsy; ^†^ Micrometastasis is defined as clinically detectable nodal metastases confirmed pathologically.

### 4.3. Step 3: Re-Excise with a Margin

In order to ensure complete removal of the primary melanoma, it is recommended that the scar following excision of an *in situ* melanoma (including Hutchinson’s melanotic macule) be re-excised with a minimum margin of 5 mm [27]. Where this is not possible, adjuvant radiotherapy can be considered. The scar following removal of an invasive melanoma should be re-excised with a margin of between 1 and 2 cm, with the choice of excision margin determined primarily by the Breslow tumor thickness in millimeters (see Table 5 below).

**Table 5:** Guidelines for excision margins for melanoma *.

melanoma *in situ* (restricted to epidermis)—margin 5 mm
melanoma <1.0 mm thick—margin 1 cm
melanoma 1.0–4.0 mm thick—minimum margin 1 cm and maximum 2 cm
melanoma >4 mm thick—minimum margin 2 cm

* Recommended excision margins are under constant review.

Wider excision has been demonstrated to reduce the risk of local persistence/recurrence of the tumor and local metastasis but there is no evidence that a margin greater than 1 cm offers additional benefit in terms of patient survival.

### 4.4. Step 4: Adjuvant Surgery

While some uncertainty remains about specific subsets of melanoma patients, therapeutic elective lymph node resection is generally not recommended as adjuvant surgical treatment. This is because there is no evidence to suggest a survival advantage and there is significant potential surgical morbidity, including postoperative lymphoedema.

Identification of suspicious lymph nodes on clinical examination should be followed by fine-needle aspiration and ultrasound imaging, MRI or PET. If nodal metastasis is confirmed histologically, immediate complete regional lymph node dissection is recommended. Cure rates in the order of 30% may be achieved with completion lymphadenectomy for palpable disease. 

Completion lymph node dissection (CLND) following identification of micrometastasis on sentinel lymph node (SLN) biopsy is more controversial as the proportion of lymph node micrometastasis that progresses to symptomatic disease is not known. It has been suggested that early detection of occult nodal disease provides greater regional control [14]. Patients who have completion lymph node dissection for micrometastatic disease develop fewer postoperative complications compared with patients who undergo therapeutic lymph node dissection for clinically palpable disease. As discussed above the MSLT-I trial comparing completion lymph node dissection with observation revealed an increase in overall survival in patients who received CLND after detection of positive SLN. This has prompted a further trial (MSLT-II ) to evaluate the benefits of delayed CLND with immediate CLND [14].

Previous re-excision of the scar may decrease the efficacy of sentinel node biopsy because it interferes with the lymphatic drainage of the site. Almost all patients with a high-risk invasive melanoma who are considering participating in an adjuvant therapy clinical trial subsequent to re-excision of the scar will require CLND for staging. Elective lymph node dissection should not be considered appropriate surgery with the exception of biopsy proven nodal disease.

Up to 5% of patients with melanoma in lymph nodes or systemic metastasis have an occult primary melanoma. These patients should be referred for complete skin examination, including examination with a Wood’s lamp to identify regressed melanoma. Referral for ophthalmological examination can also be considered. The ability to detect the primary melanoma does not influence the management of the metastatic disease. 

### 4.5. Step 5: Adjuvant Chemotherapy and Radiotherapy

#### 4.5.1. Stage 0-IIA Disease

Standard treatment options for Stage 0-IIA disease are limited to surgical resection with lymphatic mapping and appropriate lymph node dissection as described above. In current literature, the use of adjuvant chemotherapy has not been shown to impact survival. 

#### 4.5.2. Stage IIB-III Disease

##### Interferon alpha-2b

Interferons (IFN) are potent immunomodulators that are commonly used in malignancies due to various antiangiogenic, anti-proliferative and pro-apoptotic proprieties [28,29]. Of these, high-dose interferon alpha 2b (IFN-a2b) has been the most widely evaluated for the use in the treatment of melanoma. The use of high-dose IFN-a2b has been approved in an adjuvant setting for patients with surgically resected melanoma with regional lymph node metastasis (Stage IIB to III disease). 

The initial trials by the Eastern Cooperative Oncology Group [30,31] produced robust results. On pooled analysis, an improvement in recurrence free survival was identified in patients with Stage IIB and III melanoma following the use of high-dose IFN-a2b. Subsequent comprehensive meta-analyses have illustrated the positive effects of IFN-a2b in high-risk melanoma patients. Wheatley *et al*. estimated increasing recurrence free survival benefits in patients treated with increasing IFN doses when compared to observation groups. In this initial review, the effect on overall survival remained less significant [32]. More recently, a Cochrane review of the use of IFN alpha in melanoma estimated significant reductions in the risk of recurrence (HR 0.82; 95% CI; 0.77–0.87, *p* < 0.001) and overall death (HR 0.82; 95% CI; 0.83–0.96; *p* = 0.002) [33].

Pegylated Interferfon alpha-2b is a form of immunotherapy characterized by a longer half-life with a postulated improvement in toxicity incidence. The European Organization for Research and Treatment of Cancer trial (EORTC) assessed the use of pegylated IFN in melanoma. The results from EORTC illustrated a benefit in recurrence free survival and no change in overall survival [34]. This method of treatment was approved in 2011 by the FDA for treatment of melanoma with microscopic or gross nodal involvement based on these findings.

These benefits must be balanced against considerable, but rapidly reversible, toxicity. Patients often develop significant constitutional symptoms such as severe fatigue and occasionally depression, as well as abnormal liver function tests, thyroid dysfunction and neutropenia.

#### 4.5.3. Stage IV Disease

Patients with systemic metastases require multidisciplinary specialist care and palliative care health professionals may play an integral role in the management of such disease. Surgical resection should be considered for oligometastatic melanoma in the brain, lung, liver or peritoneal cavity. Stereotactic radiotherapy has a demonstrated role for oligometastatic cerebral metastases, which are not suitable for resection. Radiotherapy is often recommended for palliation of cerebral, bone or soft tissue metastases. Isolated limb perfusion, radiotherapy or topical immunotherapy may be used to control in transit cutaneous metastases [35].

With respect to the use of chemotherapeutic agents in Stage IV disease, cytotoxic therapies have long been held as the standard of treatment, although with limited success as discussed below. Over the recent period, new systemic therapeutic agents have been shown to improve on results obtained by the previous cytotoxic agents. Such agents include BRAF and MEK inhibitors. Utilizing these agents, various single agent regimens with trametinib, and MEK and BRAF inhibitors have resulted in significant short-term survival advantage. An initial response is almost universal, but frequently short-lived. Tumor resistance to these agents is generally not reversed by substitution with a different single agent, but this may still be considered. 

The best response is seen in combination therapy with a BRAF inhibitor and a MEK inhibitor and in particular when dual therapy is used at the outset. Durable responses can be seen with ipilimumab which extend beyond 4 years

##### 4.5.3.1. Checkpoint Inhibitors

Ipilimumab is a fully human IgG1 monoclonal antibody that blocks the T cell surface protein CTLA-4 that has immunoregulatory functions [36]. A durable response can be seen with ipilimumab, more so that what targeted therapy has produced [37]. Clinical trials using ipilumimab [NCT00636168] in advanced melanoma have demonstrated an improved 1 and 4 year survival [38,39]. Despite the significant improvement in prognosis, the use of Ipilimumab is characterised by significant toxicity including rash, colitis and hepatitis. Ipilimumab was approved in March 2011, by the FDA, for metastatic melanoma [40].

Nivolumab is an antibody against the programmed cell death receptor (PD-1) that has also been shown to induce melanoma regression [41]. A 2 year survival rate of 43% was seen in one study [42]. When used concurrently in combination with Ipilumimab, the response rate was 40% [43]. One third of patients had rapid and deep tumor regression [43]. Further clinical trials are still in progress [NCT01927419].

##### 4.5.3.2. BRAF and MEK Inhibitors

Various inhibitors of BRAF kinase and MAP kinase block oncogenic signal transduction pathways. These agents are only efficacious in malignancies with reported BRAF mutation in the V600 position [44], present in 50% of superficial spreading melanoma and nodular melanoma [45]. The BRAF inhibitors have a unique toxicity profile including arthritis, photosensitivity, dermatitis, keratosis pilaris, hyperkeratotic palms and soles and the development of non-melanoma skin cancer [46,47]. SCC and keratoacanthoma may develop in 10%–25% of people treated with a median time to development of nine weeks [48,49].

Vemurafenib is an inhibitor of the oncogenic BRAF kinase [50]. It received approval by the FDA in the U.S. for metastatic melanoma in August 2011. The overall response rate is 53%, though most tumors shrink to some extent, and the median duration of response is 6.7 months [44]. In most patients, the melanoma relapses as a result of the development of alternate oncogenic pathways. There is a rationale that combination chemotherapy with ipilimumab or IL-2 may delay loss of response and relapse [51]. Phase I clinical trials combining vemurafenib and ipilimumab [NCT01400451] revealed hepatotoxicity [52]. Further studies examining combined targeted therapies and immunotherapy treatments are currently underway [53].

Dabrafenib is another BRAF inhibitor that when used as monotherapy has a similar benefit to vemurafenib [54]. Recent phase III trials [NCT01227889] suggest a significantly improved progression free survival when compared to conventional cytotoxic chemotherapeutic agents [55]. 

Various other MEK and BRAF inhibitors are currently in trial for systemic use in stage IV melanoma, such as Trametinib [NCT01245062] [56] and Selumetinib [NCT01116271] [57].

##### 4.5.3.3. Cytotoxic Chemotherapy

The standard therapy for advanced disease up until recently has been dacarbazine. Its overall partial response rate is in the order of 10% with a no proven impact on survival [58]. Temozolomide is an oral analog of dacarbazine with similar efficacy, however it more readily crosses the blood-brain barrier and has activity against brain metastases [59]. Fotemustine has demonstrated higher response rates than dacarbazine but with no improvement in overall survival, although like temozolomide appears to be active against brain metastases [60].

##### 4.5.3.4. Combination Targeted Therapies

Various combinations of targeted systemic therapies are currently subject to phase II clinical trials in the U.S. as adjuvant therapy for high-risk melanoma [57]. Combination therapy using a BRAF inhibitor with the MEK inhibitor appears to increase efficacy [61]. The proposed benefit is based on theories of prevention the development of MAPK-dependant resistance mechanisms and by reducing BRAF-inhibitor related toxicities in the nonmelanoma BRAF wild-types as discussed above [53,57].

Phase II trials in metastatic melanoma assessing the addition of a second MEK/BRAF inhibitor have produced showed a significant improvement is response rate and progression free survival when compared to monotherapy [61]. The importance of simultaneous rather than sequential therapy is highlighted by the finding that response rates to trametinib are greater in treatment naïve patients and lower in people who have had prior treatment with a BRAF inhibitor [62].

Phase III studies combining trametinib and dabrafenib in the adjuvant treatment of stage IIIC or stage IV melamona currently are recruiting patients [NCT01584648 and NCT01597908]. Sentinel lymph node biopsy should now be discussed with all patients with a high-risk invasive melanoma. Where possible, patients with primary invasive melanoma should be given the option of early review (ideally within two weeks) in a specialist multidisciplinary clinic where assessment for sentinel node biopsy, surgical re-excision of the scar and genotyping of any lymph node melanoma can be performed.

### 4.6. Step 6: Adjuvant Radiotherapy

While primary radiotherapy is occasionally used for unresectable lentigo maligna or invasive melanoma, it is more commonly used as adjuvant radiotherapy for cutaneous melanoma likely to recur locally. Radiotherapy is not advised for local control of inadequately excised tumors—instead every effort should be made to achieve good margins.

Adjuvant radiotherapy is also used to prevent recurrence following regional lymph node resection. Common indications include more than three nodes with metastasis, a large tumor mass in a single node, or extracapsular spread. There is no data to suggest that adjuvant radiotherapy improves overall survival [63]. Radiation can reduce relapse free survival, but in some cases it results in lowered quality of life due to lymphedema [64]. Significant consideration should be given to the lymphedma risk when planning for adjuvant radiation, and the anticipated benefit in reducing relapse in the locoregional area should outweigh the projected lymphedema risk.

### 4.7. Step 7: Follow-Up Examination for Metastasis and Subsequent Primary Melanoma

All patients diagnosed with invasive melanoma require periodic follow-up that includes examination and palpation for local recurrence, in transit metastasis, lymph node metastasis and hepatomegaly. Additional examination and investigation should be guided by reported symptoms. 

For the 90% of Australians [65] who survive melanoma, there is a nine-fold increased risk of developing a subsequent primary melanoma [66]. This risk is also influenced by other risk factors including family history, skin type, hair color and the presence of significant solar skin damage including non-melanoma skin cancer. It is recommended that all melanoma patients, including those with *in situ* melanoma, have a complete skin check at least once a year for life following the diagnosis of melanoma. 

As there is a familial tendency to melanoma, all first-degree relatives of a patient diagnosed with melanoma should be encouraged to go for a full skin examination to identify a previously unsuspected melanoma and to assess risk of future development of melanoma. Relatives assessed to be at high risk should be offered a surveillance program. 

### 4.8. Step 8: Lifestyle Modifications to Reduce Risk of Metastasis and Subsequent Primary Melanoma

Melanoma in a pregnant woman should be treated no differently to melanoma in a non-pregnant woman. Termination of a pregnancy may be considered if there is a pressing need to start radiotherapy or chemotherapy with traditional agents that will harm the fetus. HRT or oral contraceptives are not contraindicated in women who have had a melanoma. It is common to advise women to avoid pregnancy for two years after apparent successful treatment of a high-risk melanoma during pregnancy, in accordance with the risk of metastatic recurrence.

While melanoma is popularly attributed to UV-B radiation, the actual UV action spectrum that causes melanoma is unknown. A number of animal models recently identified UV-A as important in melanoma induction [66]. In view of this as well as the systemic immunosuppression associated with UV-A exposure, it is prudent to recommend melanoma patients avoid solaria (that predominantly emit UV-A) and take precautions to minimise solar UV exposure. The “Slip, Slop, Slap” message (*i.e.*, slip on a shirt, slop on some sunscreen and slap on a hat) should be reinforced to all melanoma patients. Some patients may require serial serum vitamin D estimation and oral vitamin D replacement if deficient. Sunscreens generally block UV-B better than UV-A but recent changes in sunscreen standards should result in better UV-A blockage.

When first-degree relatives of the melanoma patient attend for their skin check, they should also receive advice regarding both primary and secondary prevention of melanoma. In particular, they should be advised to avoid solaria and take precautions to minimise solar UV exposure by employing the Slip, Slop, Slap technique. Furthermore, they should be educated on the importance of an early detection of melanoma and advised to seek medical assistance immediately should they suspect the development of a melanoma.

There is a growing body of evidence that the patients with melanoma are also susceptible to other malignancies. While the risk of other solar-induced skin cancers is obvious, the relative risk associated with various internal malignancies is less obvious.

These risks should be assessed in conjunction with any family history of internal malignancy, smoking and other cancer risk factors to determine whether any additional screening is appropriate.

### 4.9. Step 9: Counseling

Patients with invasive melanoma and their families will have complex social, psychological and financial issues. Life insurance and income protection insurance may be declined even for people with thin melanomas. The nature and intensity of these issues will vary from person to person and with disease severity. Psychologists with experience in palliative care should be involved when the patients’ needs and those of the family can no longer be met by the treating physician.

In our clinic, all patients are encouraged to make contact with a psychologist soon after diagnosis to establish a relationship should help be required subsequently. Many patients with ordinary curable melanoma benefit from this service

## 5. Conclusions

Excluding non-melanoma skin cancer, melanoma is now the third most common cancer in Australia. Up until 2010, the gradual reduction in mortality has occurred as a direct result of improved rates of early diagnosis without concomitant improvement in the survival of advanced disease. 

Systemic therapy for stage IV melanoma that is BRAF (V600E) mutation positive is rapidly emerging and combination therapy with a BRAF and MEK inhibitor show particular promise [NCT01584648] with the median progression free survival reported as 9.4 months in patients with combination therapy compared to 5.8 months in those receiving dabrafenib monotherapy [61]. Unfortunately these agents are not suitable for BRAF-negative tumors, are not curative and most patients ultimately relapse and die. For BRAF positive or negative tumors anti CTLA-4 and anti-PD-1 antibodies show promise. The role of these agents as adjuvant therapy to improve survival for high-risk melanoma patients is potentially of even greater significance and is currently being investigated.

These agents are expensive and consideration of enrolment of patients in appropriate clinical trials remains a high priority.

In order for melanoma patients to be eligible to participate in clinical trials, patients diagnosed with invasive melanoma should be referred to a specialist multidisciplinary clinic for staging, consideration of surgical re-excision of the scar, options regarding adjuvant therapy and consideration of appropriate therapy for metastatic disease.
